# Dietary Determinants of Renal Calculi: A Case-Control Study From a Tertiary Care Hospital of Western Rajasthan

**DOI:** 10.7759/cureus.31460

**Published:** 2022-11-13

**Authors:** Sonali Bhattacharya, Nitin K Joshi, Yogesh K Jain, Nitin Bajpai, Pankaj Bhardwaj, Manish Chaturvedi, Manoj S Patil, Abhay Gaidhane, Zahiruddin Quazi Syed, Deepak Saxena

**Affiliations:** 1 School of Public Health, All India Institute of Medical Sciences, Jodhpur, IND; 2 Department of Community Medicine & Family Medicine and School of Public Health, All India Institute of Medical Sciences, Jodhpur, IND; 3 Nephrology, All India Institute of Medical Sciences, Jodhpur, IND; 4 Research and Development, Jawaharlal Nehru Medical College, Datta Meghe Institute of Medical Sciences, Wardha, IND; 5 School of Epidemiology and Public Health, Jawaharlal Nehru Medical College, Datta Meghe Institute of Medical Sciences, Wardha, IND; 6 Community Medicine, Jawaharlal Nehru Medical College, Datta Meghe Institute of Medical Sciences, Wardha, IND; 7 Public Health, Indian Institute of Public Health, Gandhinagar, IND; 8 Public Health, Jawaharlal Nehru Medical College, Datta Meghe Institute of Medical Sciences, Wardha, IND

**Keywords:** logistic models, rajasthan, india, dietary, risk factors, kidney calculi

## Abstract

Introduction

Nephrolithiasis affects all countries of the world with an approximate global lifetime prevalence of 15-20%. In India, 12% of the total population is anticipated to have renal stone disease. This study was aimed at providing a relationship between various dietary factors in the formation of renal stones.

Methods

A case-control study was conducted among 207 patients (106 cases and 101 controls) attending the outpatient and inpatient departments of a tertiary care hospital in Jodhpur, Rajasthan. All the participants with confirmed renal stones by means of ultrasound and radiographic evaluation, aged 15-65 years were included as cases and were matched on age and gender with controls. Pearson chi-square test followed by binary logistic regression was used to assess significant associations.

Results

Out of all participants, 71.0% were males and 65.7% were from the age group 41-65 years. The study showed a statistically significant association between renal stones and high salt intake, reduced water intake, less consumption of milk and milk products, daily intake of tea, consumption of oxalate-rich foods and consumption of junk foods.

Conclusion

Dietary factors play an important role in the risk of the development of renal stones. Simple dietary modifications may significantly reduce the chances of the development of nephrolithiasis, especially in the vulnerable population.

## Introduction

Kidney stone disease (KSD), also known as renal stone disease, nephrolithiasis or renal calculi, is a serious disorder of public health concern. Renal calculi are crystalline structures formed when the concentration of ions and solutes such as H+, Na+ and uric acid are present in higher-than-normal amounts in a state known as supersaturation. Such states are a result of multi-factorial epidemiological and biochemical risk factors, such as diet, age, race, obesity, co-morbidities, physical activity, temperature, genetics and medications [1-3]. Kidney stones have the potential to cause serious discomfort and can impair the normal functioning of the kidney [4]. As the size of renal calculi increases it causes serious discomfort, and if neglected can lead to considerable harm and may produce several types of kidney damage. Furthermore, the treatment of kidney stones is very costly, requiring pharmacological and surgical interventions [5,6].

Over two million people are affected by renal stone disease as per the World Health Organisation with a global incidence of 3-5% annually and a lifetime prevalence of 15-20% [2,4]. The incidence of renal stones in developing and developed countries is increasing at an alarming rate. The recurrence rate of renal stones is 10% after the first year, 35% after the fifth year, 50% after the 10th year, and 75% after the 20th year of stone formation [4]. In India, KSD affects approximately two million people every year. Parts of Gujarat, Maharashtra, Rajasthan, Delhi, Haryana, Punjab and some parts of North Eastern states have been declared as the stone belt of India [1]. Out of the total Indian population, 12% is reported to be prone to renal stones and of this 12%, 50% are severely affected by renal damage, resulting in loss of kidneys [3].

Rajasthan state, because of its hot climate and higher tendencies for an oxalate-rich diet, animal protein, high salt (NaCl) intake and decreased fibre intake has a high incidence of renal stones [7-9]. Treatment of renal stones imposes a high financial burden and prevention of nephrolithiasis can have a significant impact in reducing the financial burden [10,11]. Due to various associated complications and multifactorial causation [12], understanding the etiology of renal stones is necessary for their prevention. Therefore, this study was conducted to establish the relationship between dietary factors and the formation of renal stones.

## Materials and methods

A case-control study was conducted to include patients attending the outpatient and inpatient departments of a tertiary care hospital in the months of September and October 2020. Matching of cases and controls was done on the basis of age and gender. The setting for the study was a tertiary care hospital with super specialty facilities in Rajasthan. According to diagnostic criteria, all the participants with confirmed renal stones by means of ultrasound and radiographic evaluation and aged 15-65 years were included as cases. Controls in the study were the patients attending Urology and Nephrology OPD from the same tertiary care hospital who did not have a renal stone in the same period.

Data was collected after the Institutional Ethics Committee of All India Institute of Medical Sciences (AIIMS) Jodhpur issued approval AIIMS/IEC/2020-21/3012, administrative permission, and informed consent from participants. A list of 121 cases and 128 controls was obtained from the department which were to be interviewed, out of which only 106 cases and 101 controls responded to our calls. Reasons for non-response included: not reachable phone number, long duration of the interview, non-receiving of calls, death of the patient, etc. Data was collected by the investigator with the help of telephonic interviews with the patients. In cases where the patient was not able to answer the calls, their family members were interviewed regarding the patient's diet. The patients were interviewed on their dietary habits. There was no defined recall period. Only the period before the diagnosis of renal stones was considered. All the questions of the questionnaire were covered and each interview took around 20-25 minutes. It took 40 days to complete the interview of all the cases and controls. A semi-structured demographic proforma and semi-structured questionnaire on risk factors of renal stones was used to collect data from participants regarding aspects of lifestyle, family history, dietary intake with frequency, and existing co-morbidities. The content validity of the tool was established with the help of expert opinion.

The obtained data were coded and analyzed by using SPSS version 20 (IBM Corp., Armonk, NY, USA). Demographic characteristics of participants were analyzed using descriptive statistics i.e., frequency and percentage. To find an association between risk factors and renal stones, inferential statistics were used. The goodness of fit statistics was assessed using Pearson chi-square followed by binary logistic regression. Interpretation of data was done by considering odds ratio (OR) and 95% confidence interval with p-value <0.05 considered to be significant.

## Results

A total of 207 study participants who visited the Department of Urology and Nephrology (106 cases and 101 controls) were included in the study as per the inclusion criteria. Out of these, 65.7% (n = 136) were in the age group of 41-65 years and 71.0% (n = 147) were males. Most of the participants were employed (n = 136; 65.4%) and 28.5% (n = 59) were professionals, while, 21.3% (n = 44) were illiterate, 16.4% (n = 34) were educated to primary level, 12.1% (n = 25) to middle school and 21.7% (n = 45) to high school. 89.9% (n = 186) participants were married and the majority (n = 129; 62.3%) had normal BMI (Table 1).

**Table 1:** Frequency and percentage distribution of the study participants based on demographic variables

Sample characteristics	Cases (n = 106)		Controls (n = 101)	
	Frequency	Percent	Frequency	Percent
Gender				
Male	73	68.9	74	73.3
Female	33	31.1	27	26.7
Age in years				
Age group 15-40	39	36.8	32	31.7
Age group 41-65	67	63.2	69	68.3
Marital status				
Unmarried	10	9.4	16	15.8
Married	96	90.6	85	84.2
Employment				
Unemployed	36	34.0	29	28.7
Employed	70	66.0	72	71.3
Level of education				
Illiterate	24	22.6	20	19.8
Primary	15	14.2	19	18.8
Middle school	10	9.4	15	14.9
High school	21	19.8	24	23.8
Professional	36	34	23	22.8
BMI				
Optimum BMI (18.5-24.9)	66	62.2	63	59.4
Not optimum BMI (<18 and >25)	40	39.6	38	37.6

A total of 15 risk factors were studied and Chi-square was performed to determine the relationship between risk factors and the formation of renal stones. Significant values were found associated with low water intake of less than 2 litres/day (χ2 = 9.39, df = 1, p = 0.002), intake of oxalate-rich fruits and vegetables more than three times per week (χ2 = 4.65, df = 1, p = 0.031), consumption of junk food (artificially sweetened beverages and packed food) at least once a week (χ2 = 7.56, df = 1, p = 0.006), non-consumption of milk products at least once a day (χ2 = 7.56, df = 1, p = 0.006), daily consumption of tea (χ2 = 4.13, df = 1, p = 0.042) and intake of extra salt with meals (χ2 = 10.78, df = 1, p = 0.001). No significant associations were seen between risk of stone formation and age, gender, type of diet (vegetarian/non-vegetarian), smoking, alcohol consumption, history of urinary tract infection, source of drinking water, body mass index or consumption of nuts and seeds (Table 2).

**Table 2:** Association between risk factors and renal stones

Sample characteristic	Cases (n=106)	Controls (n=101)	χ^2 ^value	Degrees of Freedom	p-value
Age					
15-40	39	32	0.59	1	0.439
40-65	67	69			
Gender					
Male	73	33	0.46	1	0.58
Female	73	27			
Water intake					
Less than 2 L/day	49	26	9.3926	1	0.00217
More than 2L/day	67	75			
Salt intake					
Extra salt	42	19	10.7768	1	0.0010
No extra salt	64	82			
Smoking					
Yes	22	16	0.7749	1	0.378694
No	84	85			
Alcohol					
Yes	11	7	0.7375	1	0.39047
No	95	94			
Urinary Tract Infection					
Yes	24	21	.0632	1	0.80153
No	82	80			
Diet					
Non- vegetarian	30	29	.0117	1	0.91398
Vegetarian	76	72			
Milk products					
Not daily	67	45	7.5567	1	0.0059
Daily	39	56			
Tea					
Daily	58	41	4.132	1	0.042
Not daily	48	60			
Oxalate rich fruits and vegetables					
At least once a week	77	29	4.6446	1	0.0311
less than once per week	59	42			
Nuts and seeds					
At least once a week	48	36	1.993	1	0.1580
Less than once per week	58	65			
Junk food					
At least once per week	61	42	7.5567	1	0.0059
Less than once per week	45	59			
Source of water					
Filtered	48	38	1.4255	1	0.2323
Other sources	58	63			
Body Mass Index					
Not optimum BMI	40	38	0.0003	1	0.9867
Optimum BMI	66	63			

A binary logistic regression was computed to find the adjusted odds ratio based on risks identified with Pearson’s Chi-square test. It was observed that the intake of a high amount of salt (OR = 2.70, p = 0.006) and low intake of water (OR = 2.40, p = 0.010) have an association with the risk of developing renal stones. Conversely, daily consumption of milk and milk products (OR = 0.380, p = 0.003) was found to be associated with reduced risk of renal stone development (Table 3). 

**Table 3:** Logistic regression analysis of risk factors for renal stone disease

Risk factors	Df	OR	95% Confidence Interval		Significance (p)
			Upper limit	Lower limit	
Smoking	1	1.137	3.005	0.430	0.796
Alcohol	1	0.985	3.773	0.257	0.983
BMI	1	0.899	1.709	0.472	0.744
Diet (veg/nonveg)	1	0.916	1.880	0.446	0.811
Oxalate rich fruits and vegetables	1	1.793	3.487	0.922	0.085
Nuts & seeds	1	1.817	3.482	0.949	0.072
Daily intake of milk/milk products	1	0.380	0.721	0.201	0.003
Daily intake of tea	1	1.746	3.302	0.923	0.086
Junk foods	1	1.333	2.498	0.711	0.370
Salt intake	1	2.702	5.515	1.324	0.006
Water intake	1	2.391	4.626	1.236	0.010
Source of water	1	1.043	1.959	0.555	0.897
Urinary tract infection	1	1.081	2.268	0.515	0.837

## Discussion

This study aimed to determine the association between dietary and environmental factors in the formation of renal stones. Findings of the present study revealed that most of the participants were in the age group of 41-65 years and were males. Similar findings were revealed by a study done by Manzoor et al. (2017) in Karnataka to find out the risk factors for renal stones reporting a greater prevalence in men suffering from renal stones as compared to women [13]. Globally prevalence of renal stones increases as age advances with the peak ranging from 40-59 years as evident from the study conducted by Romero et al. (2010) [14].

Another finding from the study was that the odds of developing renal stones are 2.7 times higher in those who consume high amounts of salts. Such findings are supported by evidence generated by studies such as those of Massey et al. (1995) in a review of salt-loading studies and reports of free-living populations in Cappuccio et al. (2000) through a review of animal, clinical and epidemiological studies [15,16]. This may be attributable to an aggravation of urinary calcium excretion from the body as a result of high salt intake, which in turn leads to renal calculi.

Furthermore, a negative association was observed between the consumption of milk and milk products and the occurrence of renal stones, inferring that participants who consumed milk products daily were less likely to develop renal stones. The finding is supported by studies revealing lower dietary calcium as an established risk factor for kidney stone disease by Taylor et al. (2013) and Sorensen (2014) since more calcium in the intestinal lumen results in lower intestinal absorption of oxalates and lower urinary excretion of calcium [17,18].

Reduced water intake was also found to be associated with a 2.3 times greater risk of renal stones. Increased water intake dilutes the urine, thus decreasing the concentration of acids and supersaturation of calcium oxalates. This finding was supported by Mitra et al. in a study conducted in West Bengal amongst 1,266 patients, Clark et al. (2016) in a review of chronic kidney disease progression and Littlejohns et al. (2019) in a population-based prospective cohort study in the UK Biobank [19-21]. Moreover, Siener et al. (2005) revealed that low fluid intake along with an increased intake of alcohol and protein were the most important risk factors for renal stone formation [22].

Significant associations were also observed between the intake of junk food as well as tea consumption and a higher risk of nephrolithiasis, as reported in other studies such as by Ferraro et al. (2013) amongst 194,095 participants and Wu et al. (2017) amongst 9,078 Northern Chinese adults [23,24].

Public health implications of the research

Northwest Rajasthan is one of the stone belts of India. Renal calculi are a rising concern and are associated with hematuria, chronic renal disease, renal failure, heart disease, and hypertension. The incidence of renal stones is higher in Rajasthan due to lifestyle habits such as salty food preparations and occupational reasons such as farming which increases the risk of dehydration which in turn leads to renal stones. This study helps in ruling out the dietary determinants of renal calculi. A promising solution to the increased risk of renal calculI can be dietary modifications which will help in better preventive and promotive care of the patients.

## Conclusions

Nephrolithiasis remains a serious disorder that affects all regions of the world. Dietary factors play an important role in the risk of the development of renal stones. Understanding the associated risk factors is of utmost importance. Our study shows factors such as salt and water intake, age group, milk products and junk food are associated with kidney stone formation. Understanding the associated risk factors is of utmost importance. Simple modifications such as low salt intake, less tea consumption, lowering junk foods intake, consumption of more than 2 litres of water per day, consuming fruits and vegetables with low oxalate and daily consumption of milk/milk products in the diet may significantly reduce the chances of development of nephrolithiasis, especially in the vulnerable population in terms of gender and age.

## Appendices

QUESTIONNAIRE

Demographic profile

1.     Date   ____________

2.     Name of the patient   _______________________________

3.     AIIMS patient ID______________________________________________

4.     Age   ____________

5.     Gender: M/F

6.     Address   _____________________________________________________________

7.     Phone No   ___________________

8.     Marital status _____________

9.     Educational qualification    _________________

10.  Eemployed:     Yes/NO

11.  No. of Family members:

12.  Details of family members having kidney stones, if any:

B. Information Regarding Kidney Stones (for cases)

1.     Location  and size of stone:   

a. Left kidney________________b. Right kidney_____________ 

2.     Type of stone _______________________________________

3.     What made you approach the doctor?

a.     (a)Symptoms:

Renal colic/Hematuria/Nausea/ Vomiting/ Burning on urination   /Abdominal distention /Reduced urination/Less no. of voids per day/Loss of appetite/Loss of weight/Pain/Any other __________________________________________

b.     During other health check up

4.     How many times have you undergone surgery for removal of kidney stones? _______________________________________________________________

5.     Which treatment did you under take?

i. Drugs           ii. No treatment yet     iii. Surgery

6.     How much time did it take to recover from operation/complication _______________________________________________________________

7.     Have you ever gone for other treatments? Yes/No

If yes, Ayurvedic medicine/Homeopathic medicine/Any other

_______________________________________________________________

C. Information regarding other complications/disorders and risk factors

1.     Other complications Do you suffer from any other complication?  Yes/No

a.     If yes, which one? Obesity/Diabetes/Hormonal imbalance/High blood pressure/ Urinary Tract infection/ Gastric acidity/ Gout/ Calcium deficiency/ any other __________________________________________

b.     If no, did you suffer from any of the above disease in the past?  Yes/No

____________________________________________________________

D. Personal habits

Do you smoke?  Yes/No

c.     Frequency per day__________

d.     Do you drink alcoholic beverages?  Yes/No

How often and in what quantity?

i. Daily

ii. Weekly

iii. Monthly

iv. Occasionally

E. Anthropometry

Height (in cm):

Weight (in kg):

F. Dietary assessment

1. Frequency of meals taken per day: Twice /Thrice/More

2. Regularity of taking meals Regular/Irregular

3. General mode of taking meals Small/moderate/heavy

4. Type of diet: vegetarian/Non vegetarian

Food Frequency Table (Table 4)

**Table 4:** Food Frequency Table

Food stuffs	Frequency of consumption				
	Daily	Alternatively	Weekly	Monthly	rarely
Cereal products					
Oxalate rich fruits and vegetables					
Nuts and seeds					
Milk and milk products					
Animal products					
Beverages					
Junk food					

7. Your preference about salt consumption in meals More salted/Moderate/Low

8. How much water you drink every day:  <1L/1-2L/2-3L/>3L

9. You are using water from: filter/other

10. Are you allergic to any food stuff? Yes/No

If yes, then to which food? __________________________

11. Have you ever been diagnosed, hospitalized, had surgery, or been evaluated in the emergency room for Urinary Tract Infection? Yes/No

12. Any additional information ___________________________________________
